# Comparative genomic analysis of carbon and nitrogen assimilation mechanisms in three indigenous bioleaching bacteria: predictions and validations

**DOI:** 10.1186/1471-2164-9-581

**Published:** 2008-12-03

**Authors:** Gloria Levicán, Juan A Ugalde, Nicole Ehrenfeld, Alejandro Maass, Pilar Parada

**Affiliations:** 1 Biosigma 'S.A.', Loteo Los Libertadores, Lote 106, Colina, Chile; 2 Laboratory of Bioinformatics and Mathematics of the Genome, Center for Mathematical Modeling, Faculty of Mathematical and Physical Sciences, Avda Blanco Encalada 2120, 7th Floor, University of Chile, Santiago, Chile; 3 Department of Mathematical Engineering and Center for Mathematical Modeling (UMI 2807, CNRS), Faculty of Mathematical and Physical Sciences, Avda Blanco Encalada 2120, 7th Floor, University of Chile, Santiago, Chile; 4 Biology Department, Chemistry and Biology Faculty, University of Santiago of Chile, Avda. Libertador Bernardo O'Higgins 3363, Estación Central, Santiago, Chile; 5 Scripps Institution of Oceanography, University of California San Diego, La Jolla, CA 92093-0208, USA; 6 Austral Biotech, Francisco Noguera 41, Piso 3, Providencia, Santiago, Chile

## Abstract

**Background:**

Carbon and nitrogen fixation are essential pathways for autotrophic bacteria living in extreme environments. These bacteria can use carbon dioxide directly from the air as their sole carbon source and can use different sources of nitrogen such as ammonia, nitrate, nitrite, or even nitrogen from the air. To have a better understanding of how these processes occur and to determine how we can make them more efficient, a comparative genomic analysis of three bioleaching bacteria isolated from mine sites in Chile was performed. This study demonstrated that there are important differences in the carbon dioxide and nitrogen fixation mechanisms among bioleaching bacteria that coexist in mining environments.

**Results:**

In this study, we probed that both *Acidithiobacillus ferrooxidans *and *Acidithiobacillus thiooxidans *incorporate CO_2 _via the Calvin-Benson-Bassham cycle; however, the former bacterium has two copies of the Rubisco type I gene whereas the latter has only one copy. In contrast, we demonstrated that *Leptospirillum ferriphilum *utilizes the reductive tricarboxylic acid cycle for carbon fixation. Although all the species analyzed in our study can incorporate ammonia by an ammonia transporter, we demonstrated that *Acidithiobacillus thiooxidans *could also assimilate nitrate and nitrite but only *Acidithiobacillus ferrooxidans *could fix nitrogen directly from the air.

**Conclusion:**

The current study utilized genomic and molecular evidence to verify carbon and nitrogen fixation mechanisms for three bioleaching bacteria and provided an analysis of the potential regulatory pathways and functional networks that control carbon and nitrogen fixation in these microorganisms.

## Background

The employment of microorganisms for metal recovery from low-grade ores and mineral concentrates and secondary materials, has developed into a successful and expanding area of biotechnology. In association with this interest, microbial communities of extreme acidophilic prokaryotes from bioleaching environments have long been the subject of active research; however, the components and interactions within these microbial communities' remains poorly understood. Recent acquisition of genomic data directly from organisms living in naturally extreme environments [1-4] in combination with genome sequencing projects of individual species [5,6] provides a novel opportunity for prediction and exploration of the metabolic details that control both individual microorganisms and microorganism communities.

Acidophilic prokaryotes involved in metal recovery from sulfide minerals include members of the Bacteria and Archaea domains. Three species of chemolithotrophic bacteria are mainly involved: *Acidithiobacillus ferrooxidans*, *Acidithiobacillus thiooxidans *and *Leptospirillum sp*., all of which obtain energy primarily from iron and/or sulfur oxidation. *A. ferrooxidans *is capable of oxidizing reduced sulfur compounds and Fe^2+ ^ions to form sulfate and Fe^3+^, respectively [7-10]. *A. thiooxidans *can only oxidize reduced sulfur compounds such as thiosulfate, tetrathionate, metal sulfides and elemental sulfur to form sulfate [7-9,11]. *Leptospirillum *sp. is solely capable of oxidizing Fe^2+ ^ions to form Fe^3+ ^[12]. These autotrophic microorganisms utilize the energy and reducing power derived from iron and/or sulfur oxidation for several metabolic processes, including CO_2 _fixation and acquisition of several sources of nitrogen. In both *Acidithiobacillus *species, CO_2 _fixation occurs via the Calvin-Benson-Bassham cycle [5,13,14] whereas *Leptospirillum *sp. grows autotrophically; however the molecular mechanisms involved in carbon fixation remain obscure.

In acidic bioleaching environments, dissolved inorganic carbon can reach levels below atmospheric concentrations average. Therefore, it is not surprising that CO_2 _concentrating mechanisms have been identified in autotrophic prokaryotes present in such environments [15,16]. In *A. ferrooxidans *(ATCC 23270), the presence of carboxysomes has been inferred from genome annotation [17], but the physiological role of this compartment and characterization of global CO_2 _concentrating mechanisms in bioleaching bacteria are yet to be determined.

Nitrogen plays an important role in the ecology of microbial communities. Therefore, understanding the molecular mechanisms involved in nitrogen fixation and assimilation are critical to understand how microorganisms adapt themselves to changes in environmental nitrogen. Ammonium, nitrate, nitrite and glutamine are the main nitrogen sources used by microorganisms in natural environments. Under low nitrogen levels, diazotrophic bacteria can fix atmospheric nitrogen under anaerobic or microaerobic conditions through the action of the nitrogenase complex. Because reduction of N_2 _to ammonium is an energy-demanding process and because the nitrogenase enzyme is very sensitive to oxygen, biological N_2 _reduction is a tightly regulated process [18,19].

The capability of microorganisms to fix atmospheric nitrogen plays an important role in recycling scarce nitrogen existing in nutrient-poor acidic conditions; however, the availability of nitrogen and the energy required for its fixation may limit bacterial growth and adversely affect the efficiency of bioleaching operations. The study of nitrogen metabolism in members of microbial communities is therefore of both fundamental and applied interest. In bioleaching communities N_2 _fixation has been predicted for *A. ferrooxidans *[5,20-22] and members of groups I [23,4] and III [24] of the *Leptospirillum *genus. Genomic analysis of these bacteria revealed the presence of genes involved in N_2 _fixation (*nif*), ammonium transport (*amt*) and genes encoding the regulatory proteins NtrC and NifA (specific activators of *nif *genes). Genes encoding the regulatory PII protein, which plays a controlling role in the nitrogen metabolism coupled to the central carbon metabolism [25,26], have also been identified.

Although carbon and nitrogen fixation has been predicted for *A. ferrooxidans *and members of the *Leptospirillum *genus, the physiology and regulation of these processes are still poorly understood. Here we report a comparative genomic analysis of the carbon and nitrogen metabolism carried out on three sequenced bacterial genomes (*A. ferrooxidans*, *A. thiooxidans *and *Leptospirillum *group II) isolated from naturally extreme environments in the north of Chile.

## Results and discussion

### Molecular mechanisms involved in CO_2 _fixation

#### CO_2 _fixation by the Calvin-Benson-Bassham (CBB) cycle

CBB is composed of 13 enzymatic reactions, 12 of which are involved in regeneration of ribulose 1,5-bisphosphate (RuBP) and one of which is responsible for CO_2 _fixation catalyzed by ribulose 1,5-bisphosphate carboxylase/oxygenase (Rubisco). The key CO_2 _fixation enzymes in the CBB cycle are Rubisco, Phosphoribulokinase (PRK) and Sedoheptulose 1,7-bisphosphatase (SBP) [27]. We searched in the genomes of *Acidithiobacillus ferrooxidans*, *Acidithiobacillus thiooxidans *and *Leptospirillum ferriphilum *for genes encoding these CBB enzymes (See additional file 1: CarbAsilProts.csv for the list and sequence of these proteins). PRK and SBP genes were identified in the *Acidithiobacillus *strains as single copies but not in the *Leptospirillum *strain. We identified canonical forms of Rubisco encoded in the genomes of both *Acidithiobacillus *species inspected. In *A. ferrooxidans *DSM 16786, two gene copies encoding Rubisco form I (*cbbSL1 *and *cbbSL2*) and one copy encoding Rubisco form II (*cbbM*) were identified, as described previously [28], whereas in *A. thiooxidans *DSM 17318, Rubisco forms I and II were each encoded by one gene. The presence of multiple sets of genes for Rubisco is well documented in the literature. For example, in *Hydrogenovibrio marinus *it has been proposed that the expression of three Rubisco genes (two of form I and one of form II) is dependent on the environmental CO_2 _concentration and that there is an interactive regulation among these genes [29]. It is interesting to note that both *Acidithiobacillus *strains examined in this study contain a *cbbR *gene upstream of the *cbbSL1 *and *cbbM *genes. CbbR is a positive regulator of *cbb *operon that coordinates the expression of three Rubisco genes [29-31]. It is likely that the presence of multiple forms and gene copies of Rubisco and a controlled Rubisco expression system allow these bacteria to rapidly respond to environmental changes in the CO_2_/O_2 _concentrations.

In the *Leptospirillum *DSM17947 strain only the non-canonical Rubisco-like protein (RLP) was identified, which is similar to Rubisco form IV of the photosynthetic thermophilic purple sulfur bacteria *Chromatium tepidum *[32]. RLP has not been demonstrated to catalyze CO_2 _fixation and therefore the enzyme may not take part in the CBB cycle [33].

The genomic analysis performed on these three genomes suggested that the two γ-proteobacteria strains from the *Acidithiobacillus *genus (strains DSM 16786 and DSM 17318) can fix CO_2 _via the CBB cycle. This is based on the identification of all 13 genes encoding enzymes required for this cycle; however, distinct forms and copy numbers of the Rubisco genes were identified. We did not identify genes encoding the key CO_2 _fixation enzymes of the CBB cycle in *Leptospirillum *DSM 17947.

#### CO_2 _fixation by the RTCA cycle

**Figure 1:**
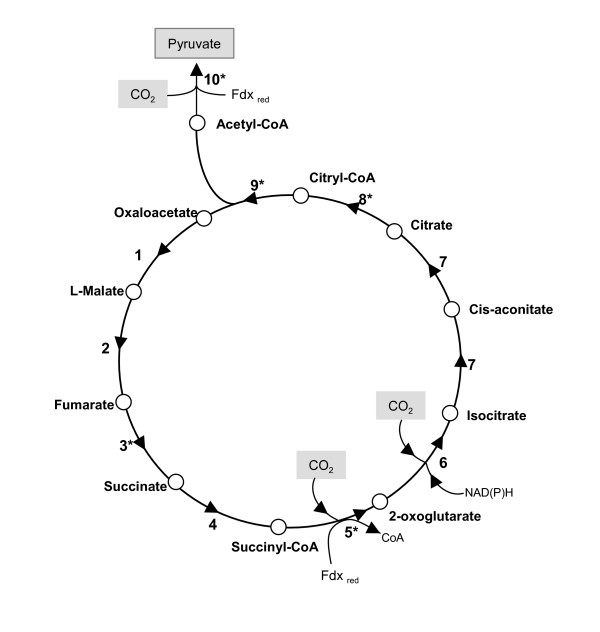
**Schematic diagram of the reductive tricarboxylic acid cycle of *L. ferriphilum *DSM 17947**. Catalytic enzymes are indicated by numbers: 1, malate dehydrogenase (EC 1.1.1.37); 2, fumarate hydratase (EC 4.2.1.2); 3, fumarate reductase (EC 6.2.1.5); 4, succinyl-CoA synthetase (EC 6.2.1.5); 5, 2-oxoglutarate ferredoxin oxidoreductase (EC 1.2.7.3); 6, isocitrate dehydrogenase (EC 1.1.1.42); 7, aconitase hydratase 1 (EC 4.2.1.3); 8, citryl-CoA synthetase (EC 6.2.1.18); 9, citryl-CoA lyase (EC 4.1.3.34); 10, pyruvate ferredoxin oxidoreductase (EC 1.2.7.1). Key enzymes are indicated by asterisks. Fdx_red_, reduced ferredoxin.

The absence of genes encoding for canonical enzymes of the Calvin cycle in *Leptospirillum *DSM 17947 suggested the existence of an alternative mechanism for CO_2 _fixation. The reductive tricarboxylic acid cycle (RTCA), which is essentially the TCA cycle running in reverse direction, also leads to the fixation of two molecules of CO_2 _and to the production of one molecule of acetyl-CoA. The acetyl-CoA formed is then reduced by carboxylation to pyruvate, from which all other central metabolites can be formed. The four key enzymes that make possible the reversal TCA cycle and pyruvate formation are ATP citrate lyase (ACL), fumarate reductase (FDR), 2-oxoglutarate ferredoxin oxidoreductase (OGOR) and pyruvate ferredoxin oxidoreductase (POR) [34,35]. The presence of such enzymes activities in autotrophically grown bacteria and archaea is considered indicative of RTCA function. Inspection of the *Leptospirillum *DSM 17947 genome showed the presence of genes coding for all enzymes of the RTCA cycle, including the four key enzymes (See additional file 1: CarbAsilProts.csv for the list and sequence of these proteins). All RTCA cycle enzymes and reactions are depicted in the metabolic scheme presented in Figure 1. The citric acid cycle moving in the forward direction, in an oxidative fashion, according to our genomic search, is unlikely, because genes encoding for 2-oxoglutarate dehydrogenase were undetected. CO_2 _fixation mechanisms other than the Calvin Cycle and RTCA cycles include the 3-hydroxypropionate and the reductive acetyl-CoA cycles [35]. No evidence of genes coding for the key enzymes of these two pathways for carbon fixation was found in *L. ferriphilum *DSM 17947.

These findings represent the first evidence of the reductive tricarboxylic acid cycle as being the autotrophic CO_2 _fixation mechanism in a member of *Leptospirillum *genus.

### Genetic characterization of *L. ferriphilum *genes encoding enzymes of the RTCA cycle

**Figure 2:**
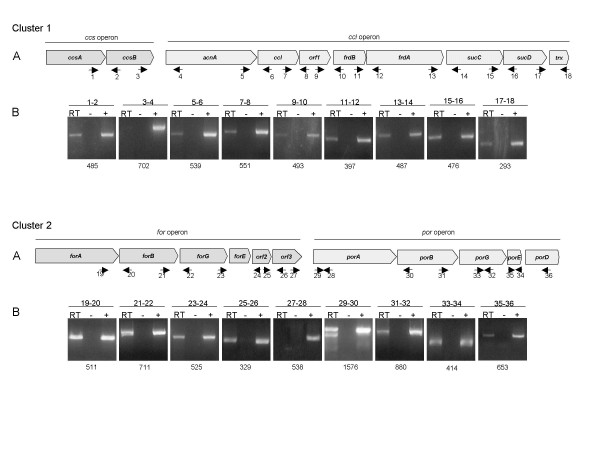
**Structure and genetic organization of the *L. ferriphilum *DSM 17947 genes in cluster 1 and cluster 2 predicted to be involved in reductive tricarboxylic acid (RTCA) cycle**. A. Schematic map of the RTCA locus. Genes located in Cluster 1: *ccsA *(citryl-CoA synthetase, subunit A), *ccsB *(citryl-CoA synthetase, subunit B), *acnA *(Aconitase A), *ccl *(citryl-CoA lyase), *orf1*, *frdA *(fumarate reductase, subunit A), *fdrB *(fumarate reductase, subunit B), *sucC *(succinyl-CoA synthetase, beta subunit), *sucD *(succinyl-CoA synthetase, alpha subunit) and *trx *(thioredoxin). Genes located in Cluster 2: *forA *(2-oxoglutarate ferredoxin oxidoreductase, alpha subunit), *forB *(2-oxoglutarate ferredoxin oxidoreductase, beta subunit), *forG *(2-oxoglutarate ferredoxin oxidoreductase, gamma subunit), *forE *(2-oxoglutarate ferredoxin, epsilon subunit), *orf2 *(hypothetical protein), *orf3 *(hypothetical protein), *porA (*pyruvate ferredoxin oxidoreductase, alpha subunit), *porB (*pyruvate ferredoxin oxidoreductase, beta subunit), *porG (*pyruvate ferredoxin oxidoreductase, gamma subunit), *porE (*pyruvate ferredoxin oxidoreductase, epsilon subunit), *porD (*pyruvate ferredoxin oxidoreductase, delta subunit). B. RT-PCR amplification (RT) of intergenic. -, RT-PCR amplification control without reverse transcriptase; +, standard PCR amplification control. Primer pairs used for amplification are indicated at the top of each panel and amplification product sizes are indicated at the bottom of each panel.

In the *L. ferriphilum *DSM 17947 genome, we detected two gene clusters, named as cluster 1 and cluster 2, encoding seven of the ten enzymes involved in the RTCA cycle (Figure 1), including the four key enzymes. The genes encoding malate dehydrogenase, fumarate hydratase and isocitrate dehydrogenase (Figure 1, reactions 1, 2, and 6) were also detected, but in a different genomic locus. In order to characterize the genetic organization of clusters 1 and 2, we examined co-transcription of these neighbor genes using a RT-PCR approach. Amplification products of the predicted size from each inter-operon region from both clusters were observed, verifying co-transcription of the four proposed operons. These results suggest that the genes contained within clusters 1 and 2 are organized into two transcriptional units (Figure 2). Genomic sequences of both clusters were inspected looking for theoretical Rho independent transcriptional terminators in the inter-operon regions, but in any of the 4 operons an evident terminator was found. Nevertheless the inter-operon regions in both cases is big enough for not to suspect a cotranscriptions of genes as it is probed by RT-PCR results (474 nts between *ccs *and *ccl *operons and 207 nts. between *for *and *por *operons. It is important to notice that our method identified three open reading frames by blast search, encoding the conserved hypothetical proteins *orf1*, *orf2 *and *orf3*, co-transcribed with the known putative RTCA cycle genes on these two clusters. Additionally, bioinformatic studies showed that each of these open reading frames contained a putative ribosome binding sequence, suggesting that they are translated as independent polypeptides.

### Bioinformatic analysis of the *L. ferriphilum *RTCA cycle predicted proteins

The RTCA cycle operates in phylogenetically diverse autotrophic bacteria and archaea [36-41]. Due to this high diversity, it is not surprising to find some differences among these organisms with respect to the catalytic mechanisms and enzymes involved in each step of the cycle. Two examples of these differences are demonstrated by conversion reactions of citrate, which form acetyl-CoA plus oxaloacetate and carboxylation of 2-oxoglutarate which forms isocitrate. Both conversions can be catalyzed in only one reaction as its described for most bacteria, or in two reactions as has been described for *Hydrogenobacter thermophilus *[42]. To elucidate the nature of the RTCA cycle enzymatic reactions for *L. ferriphilum *DSM 17947, we analyzed the deduced amino acid sequences of the predicted proteins using a bioinformatics approach.

The first key enzyme of the RTCA pathway is fumarate reductase (Figure 1, reaction 3), which catalyzes the reduction of fumarate to succinate using ubiquinol as an electron donor. This enzyme is a transmembrane protein comprised of two domains; the soluble N-terminal domain (subunit A) which is exposed to the cytoplasm and contains a covalently linked FAD, and the membrane-bound C-terminal domain (subunit B) which contains three iron-sulfur centers. Based on amino acid comparisons of these core subunits and on comparison of the metal centers and membrane anchors, fumarate reductase has been subdivided into five classes, types A-E [43]. The type E group is comprised of those enzymes that lack a canonical membrane-anchoring domain but contain amphipathic subunits that ensure interaction with the membrane. Although we searched the complete genome of *L. ferriphilum *DSM 17947 for specific sequences to each class of fumarate reductase [43], only the *frdA *and *frdB *genes were detected, which encode the flavoprotein and iron-sulfur protein subunits respectively, and were located in tandem in the *ccl *operon (Figure 2). We did not detect orthologs for any of the known membrane anchoring subunits. The FdrA and FdrB candidate proteins showed overall similarity to the corresponding subunits of *Geobacter metallireducens *(70% and 59%, respectively). In addition, FdrA has a high similarity to the SdhA subunit of succinate dehydrogenase from the cyanobacteria *Synecchocystis sp*., which resembles type E enzymes that contain a non-canonical amphipathic subunit structure [43]. Taken together, these data suggest that the fumarate reductase from *L. ferriphilum *is a new member of the fumarate reductase type E family [43]. In addition, using bioinformatics procedures, the polypeptide encoded by *orf1 *located upstream of *frdA *and *frdB *was predicted to contain four putative amphipathic helices and to be targeted towards the inner membrane of the cell. We therefore believe that *orf1 *is a candidate gene for the anchor subunit of fumarate reductase. Further research will be required to evaluate the subunit composition and the catalytic properties of the Frd enzyme from the *Leptospirillum *genus.

Another key enzyme of the RTCA pathway is 2-oxoglutarate ferredoxin oxidoreductase (OGOR) (Figure 1, reaction 5), which catalyzes the reductive carboxylation of succinyl-CoA to 2-oxoglutarate. In *H. thermophilus*, a chemolithoautotrophic hydrogen-oxidizing bacterium that fixes carbon dioxide via the RTCA cycle, two different OGOR polypeptide complexes have been reported: one with two subunits (encoded by *korAB*) and the other with five subunits (encoded by *forDABGEF*) [44]. A search of the *L. ferriphilum *DSM 17947 genome showed five similar genes to those in the *for *operon, including *forABGE*, which encode the α, β, γ, and ε subunits of OGOR (Figure 2), and two non-identical copies of *forD *(encoding the δ subunit). These latter non-identical copies of *forD *(*forD1 *and *forD2*) were identified in a different genomic loci which is strikingly different from other models described and will therefore require further analysis to confirm the nature of this putative δ subunit and its possible role in the enzymatic activity of the OGOR complex. The deduced amino acid sequences of these six OGOR subunits showed high overall similarity (66–80%) to the corresponding OGOR subunits of *H. thermophilus *and also to the conserved protein motifs common to 2-oxoacid acceptor oxidoreductases in the Prosite database (α: PF01855, β: PF02775, γ: PF01558, ε: PF00037, δ1 and δ2: PF02552). No evidence of *korAB *genes encoding a two-subunit OGOR was found.

In prokaryotes, isocitrate dehydrogenase (ICDH) (Figure 1, reaction 6) is mainly an oligomeric enzyme that catalyzes the reversible conversion of isocitrate to 2-oxoglutarate. ICDH has been mainly studied as a catabolic enzyme of TCA cycle; nonetheless, in some CO_2_-fixing organisms that utilize the RTCA cycle, special properties of the ICDH enzyme have become evident [42]. For illustration, ICDH from *Clorobium limicola *[45] is a monomeric enzyme that works more favorably fixing CO_2 _in anabolic way than in catabolic decarboxylating way, contrary to that for the ICDH enzyme from *E. coli*. In addition, a novel mechanism for efficient conversion of 2-oxoglutarate to isocitrate has been recently described for ICDH from *H. thermophilus *[46] and involves two distinct and consecutive reactions catalyzed by 2-oxoglutarate carboxylase (OGC) and oxalosuccinate reductase (OSR). In *L. ferriphilum *DSM 17947, we identified isocitrate dehydrogenase genes similar to those from *E. coli*. This suggests that *L. ferriphilum *does not utilize ICDH for anabolic CO_2 _fixation, as described for *C. limicola *or *H. thermophilus*. Experimental methods will be required to determine whether the ICDH from *L. ferriphilum *functions in a manner similar to that from *E. coli *or whether this enzyme catalyzes the reductive carboxylation of 2-oxoglutarate in a novel manner.

An important reaction in carbon metabolism is the condensation of acetyl-CoA and oxaloacetate to citrate via the TCA cycle. This reaction is catalyzed by citrate synthase; however, in the case of the RTCA cycle, the reverse reaction is catalyzed by ATP citrate lyase (ACL). ACL is a key enzyme of the RTCA cycle and is unique to organisms that utilize the RTCA cycle. ACL has also been reported in eukaryotes [47-50], where it plays an important role in supplying acetyl-CoA for fatty acid biosynthesis. ACL from *Chlorobium *[51] and fungi [52] is composed of a small and a large subunit with similarity to the N- and C-terminal half, respectively, of the mammalian single polypeptide ACL [53]. On the other hand, in the *Aquifex *and *Hydrogenobacter *genera, a novel and ACL-independent citrate cleavage pathway has been described [54,55]. In these organisms, the ATP-dependent cleavage of citrate is catalyzed by the combined action of the citryl-CoA synthetase (Ccs) and citryl-CoA lyase (Ccl) enzymes (Figure 1, reactions 8 and 9). Ccs is composed of a 46 kDa β subunit and a 36 kDa α subunit. Ccl is a single polypeptide protein of 30 kDa.

Inspection of the *L. ferriphilum *DSM 17947 genome revealed the presence of *ccsAB *and *ccl*, but not *aclAB*, suggesting that in this bacterium, citrate cleavage occurs via two successive reactions catalyzed by the enzymes Ccs and Ccl, as described for the *Aquifex *and *Hydrogenobacter *genera and for *H. thermophilus *[54,55]. In addition, the predicted polypeptide sequences of CcsA, CcsB and Ccl displayed high amino acid similarity to the corresponding proteins of *H. thermophilus *(CcsA: 74% similarity, CcsB: 75% similarity and Ccl: 66% similarity).

Pyruvate ferredoxin oxidoreductase (POR) is another key enzyme in the RTCA pathway and catalyzes the reductive carboxylation of acetyl-CoA to pyruvate (Figure 1, reaction 10). POR, like OGOR, is a member of the 2-oxoacid oxidoreductase family and both enzymes are structurally similar, making sequence comparisons difficult; fortunately, the POR and OGOR enzymes of *H. thermophilus *have been enzymatically characterized [56-59], and the amino acid sequences are available in the NCBI database. We identified five putative *por *genes (*porABGED*) in the *L. ferriphilum *genome, and based upon the similarity of their deduced amino acid sequences to those from *H. thermophilus *we assigned a putative function to these genes. The predicted proteins PorA, PorB, PorG, PorE and PorD were similar (65–79%) to α, β, γ, ε and δ subunits of the POR enzyme from *H. thermophilus *and has the conserved pattern of 2-oxoacid: acceptor oxidoreductases (Prosite database). Additionally, the POR subunit genes were clustered downstream of the *for *operon (Figure 2).

**Figure 3:**
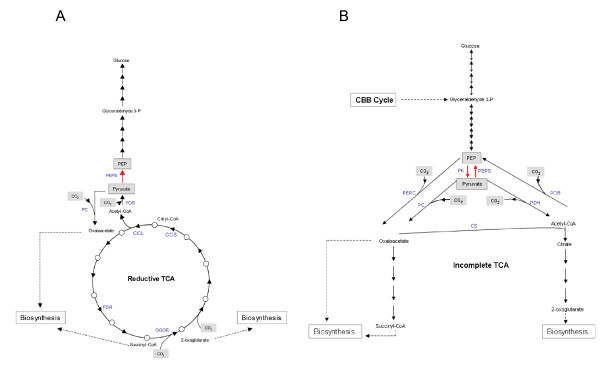
**Proposed models of the metabolic direction of the Embden-Meyerhof-Parnas (EMP) and TCA cycle pathways in the three microorganisms examined in this study**. A) Model representing the pathways utilized by *L. ferriphilum *DSM 17947. PK, pyruvate kinase (EC 2.7.1.40); PEPS, phosphoenolpyruvate syntethase (EC 2.7.9.2); PC, pyruvate carboxylase (6.4.1.1); PEPC, phosphoenolpyruvate carboxylase (4.1.1.3.1); POR, pyruvate ferredoxin oxidoreductase (1.2.7.1); PDH, pyruvate dehydrogenase (EC 1.2.4.1.); CS, citrate synthase (EC2.3.3.1).B) Model representing the pathways utilized by *A. ferrooxidans *DSM 16786 and *A. thiooxidans *DSM 17318. In *A. ferrooxidans*, the A, B and G subunits of the Por enzyme (encoded by *porABG genes*) do not have amino acid identity with those from *L. ferriphilum*. In addition, *por *genes from *L. ferriphilum *DSM 17947 were not detected in either *A. ferrooxidans *DSM 16786 or *A. thiooxidans *DSM 17318.

Pyruvate produced from the RTCA cycle is directed to gluconeogenesis (Figure 1) for the biosynthesis of several carbonated intermediate molecules required by the cell. The anabolic conversion of pyruvate to phosphoenolpyruvate (PEP) is typically catalyzed by phosphoenolpyruvate synthetase (PEPS), whereas the catabolic conversion of PEP to pyruvate is catalyzed by pyruvate kinase (PK). The combined and coordinated action of PEPS and PK allows the cell to control the interconversion of pyruvate and phosphoenolpyruvate according to its metabolic requirements. Additionally, in several organisms including bacteria and archaea, phosphoenolpyruvate diquinase (PPDK) has been reported for interconversion of these metabolites [60,61] A search of the *L. ferriphilum *DSM 17947 genome revealed a candidate gene encoding PEPS, but did not uncover any genes encoding PK or PPDK. An additional search for genes involved in glycolytic pathways revealed that *pfkA *and *pfkB*, genes encoding the catabolic regulatory enzyme phosphofructokinase, were also missing. The absence of genes for the glycolitic enzymes pyruvate kinase (PK) and phosphofructo kinase (PFK) allowed us to predict that in *L. ferriphilum*, the Embden-Meyerhof-Parnas (EMP) pathway also works preferentially in the anabolic direction. Interestingly, in *A. ferrooxidans *and *A. thiooxidans *which use the CBB cycle to fix CO_2_, genomic analysis revealed the presence of genes for both PK and PFK (PFK-2) enzymes. This suggests that in both *Acidithiobacillus *strains, the EMP pathway operates in both anabolic and catabolic directions, wherein the 3-phosphoglyceraldehyde (PGA) formed by the CBB cycle enters the EMP pathway to form glucose via anabolic reactions, or to form pyruvate via catabolic reactions. An incomplete TCA cycle can convert pyruvate to oxaloacetate as well as to succinyl-CoA and 2-oxoglutarate to form metabolites related to the biosynthesis of cellular components [62]. Hypothetical models delineating the reactions associated with the glycolytic/glucogenic pathway and the TCA coupled cycles in the *Acidithiobacillus *and *Leptospirillum *strains examined in this study are shown in Figure 3.

Our genomic analysis suggests that completely different regulatory mechanisms exist for microorganisms that fix CO_2 _via alternative mechanisms. Specifically, the *Acidithiobacillus *and *Leptospirillum *strains examined in this study fix CO_2 _by altering the direction of the central carbon metabolism.

### Molecular mechanisms involved in CO_2 _concentrating mechanisms

Carbon concentrating mechanisms are present in many species of chemolithoautotrophic bacteria, enabling them to grow in the presence of low concentrations of CO_2_. They mainly utilize bicarbonate transporters and CO_2 _traps to generate high intracellular concentrations of dissolved inorganic carbon.

Inorganic carbon transporters that deliver intracellular HCO_3 _^- ^represent an important carbon concentrating mechanism within a diverse group of microorganisms [63]. In cyanobacteria at least five distinct enzymes for active inorganic carbon uptake have been described, including BCT1 (High affinity Bicarbonate Transporter 1), SbtA (Sodium bicarbonate transporter A), BicA (Low affinity Na+-dependent Bicarbonate Transporter), NDH-1_4 _and NDH-1_3 _(NAD(P)H dehydrogenase type 1) (Reviewed in [64]). BicA is a Na^+^-dependent HCO_3 _^- ^transporter belonging to the widespread SulP (Sulphate Transporter or Permease) family [65]. In the *Acidithiobacillus *DSM 16786 and DSM 17318 strains analyzed in this work, a putative gene for the BicA transporter was identified, whereas in *L. ferriphilum *putative genes for the BCT1 transporter (*cmpABCD*) were detected.

The carboxysome is a polyhedral micro compartment located in the cytoplasm of most autotrophic bacteria and is surrounded by a proteinaceous monolayer that reportedly contains Rubisco and carbonic anhydrase (CA) [63]. CA converts accumulated cytosolic HCO_3 _^- ^into CO_2 _within the carboxysome, elevating the CO_2 _concentration in the vicinity of Rubisco [65]. Previous reports described the *A. ferrooxidans *ATCC 23270 carboxysome as being composed of at least seven peptides, all encoded by genes located in a carboxysome operon [66]. Similarly, seven candidate genes potentially involved in carboxysome formation were identified immediately downstream of the *cbbLS1 *genes in both *Acidithiobacillus *strains examined in the present report. The *cbbLS2 *and *cbbM *genes from these strains are followed by the *cbbQO *genes, which are involved in posttranslational regulation of Rubisco. Carbonic anhydrases are classified in four main forms: α-CA, β-CA, γ-CA and ε-CA [67-69]. ε-CA has been described as a novel form that corresponds to carboxysomal shell protein CsoS3 [70]. The β-CA family is comprised of enzymes from four evolutionarily distinct clades (A through D). Candidate genes for β-CA (Clade B), γ-CA and ε-CA, but not for α-CA, were identified in the *A. ferrooxidans *DSM 16786 genome. This is consistent with that reported for *A. ferrooxidans *ATCC 23270 [6]. In *A. thiooxidans *DSM17318, we only identified a single candidate gene for ε-CA, located in the putative carboxisome gene cluster. In *L. ferriphilum *DSM 17947, we identified putative genes for β-CA (clade D) and γ-CA, but not for ε-CA (encoded by the *csoS3 *gene) or for any of the carboxysome genes (*cso *genes).

Differences in CO_2 _concentrating mechanisms were also predicted from genomic analysis of the bioleaching bacteria examined herein. In both *Acidithiobacillus *strains, HCO_3 _^- ^transport via carboxysomes and the BicA-type protein was inferred. Because CO_2 _concentrating mechanisms have been described mainly among organisms that utilize the CBB cycle, such as cyanobacteria, comparative analysis with microorganisms that use the RTCA cycle is not possible. Further analysis will be necessary to determine whether *L. ferriphilum *truly lacks carboxysomes or whether an analogous structure is encoded by unidentified genes to improve the efficiency of RTCA cycle enzymes that fix CO_2_.

### Nitrogen uptake mechanisms

To gain a better understanding of the mechanisms by which the microorganisms examined herein fulfill their nitrogen requirements, we searched the genomic sequences for genes involved in the uptake of different nitrogen compounds (See additional file 2: NitAsilProts.csv for the list and sequence of proteins involved in nitrogen assimilation). From this search we identified genes that encode for the nitrogenase complex in *A. ferrooxidans *DSM 16786, genes encoding proteins involved in the assimilation of nitrate and nitrite in *A. thiooxidans *DSM 17138, and genes coding for ammonia permeases (*amtB*) in the three microorganisms.

#### Nitrogen fixation

In the *A. ferrooxidans *DSM 16786 genome, we identified a region in which genes of the nitrogenase complex and all the necessary assembly proteins are located (Figure 3), consistent with what has been reported in other strains of *A. ferrooxidans *[22]. In this region we found the nitrogenase genes *nifHDK *next to several tandem genes important for assembly of the nitrogenase MoFe cofactor (*fdxD, C1499, nifE, nifN and nifX*). Interestingly, the genes *draGT*, the products of which are associated with post-translational regulation of nitrogenase in α-proteobacteria [19,71] were situated in reverse orientation to the *nifHDK *operon. In contrast, we did not find any homologous genes in the *A. thiooxidans *DSM 17318 and *L. ferriphilum *DSM 17947 genomes, which suggests that these microorganisms utilize alternative mechanisms for assimilation of nitrogen from the environment, consistent with what has been reported for members of *Leptospirillum *Group II [2,3], and *A. thiooxidans *[5].

#### Nitrate and nitrite assimilation

**Figure 4:**
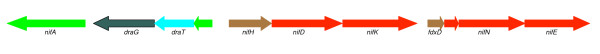
**Schematic diagram of the *A. ferrooxidans *DSM 16786 genomic region containing putative nitrogen metabolism genes**. *A. ferrooxidans *DSM 16786 genes implicated in nitrogen fixation (*nifHDK*), assembly of the nitrogenase protein (*fdxD-fdx-nifN-nifE*) and regulation of nitrogen assimilation (*nifA, draGT*) are indicated.

In addition to atmospheric nitrogen, other possible sources of nitrogen for microorganisms are nitrate and nitrite. Different nitrate and nitrite assimilation mechanisms have been described [72], but the general requirements include a transporter protein for nitrate and enzymes that catalyze reduction of nitrate to nitrite and finally to ammonia [72,73]. In *A. thiooxidans*, we found a genomic region where genes homologues to components of a nitrate and nitrite assimilation system are located (Figure 4). The elements located in this region include genes encoding a periplasmic component of a transport system (*nrtA*), a putative nitrate transporter (*narK*), the large and small subunits of nitrite reductase (*nirB *and *nirD*) and nitrate reductase (*narB*), plus a hypothetical protein conserved among several microorganisms that lacks an assigned function. The nitrogen uptake and reduction mechanisms in *A. thiooxidans *appear atypical compared to that described for other microorganisms that assimilate nitrate or nitrite [72-74]. For example, we identified a gene for the periplasmic component (*nrtA*) of an ABC transport system, but no evidence of the permease and ATPase components of this system were found. In addition, next to *nrtA *we found *narK*, which encodes a protein belonging to the major facilitator superfamily (MFS) involved in the uptake of nitrate and nitrite. We also found genes corresponding to ferredoxin-dependent reductases, which participate in nitrate and nitrite reduction [74], but again these genes were distinct from those reported for similar nitrate and nitrite assimilation systems. Specifically, genes for both nitrate reductase (*narB*), which is involved in nitrate assimilation, and for nitrite reductase (*nirBD*), which is involved in dissimilatory nitrite reduction, were identified. However, it is possible that the ammonium generated by nitrite reductase may be assimilated in *A. thiooxidans *DSM 17318.

We examined whether *A. thiooxidans *DSM17318 was capable of utilizing nitrate as the sole nitrogen source by monitoring the growth of this strain in a shaking flask experiment under aerobic conditions. Preliminary data showed that this bacterium cannot grow using nitrate as nitrogen source but can grow normally in the presence of ammonia (data not shown). Most bacteria that assimilate nitrate do so under aerobic conditions; in contrast, dissimilatory nitrate metabolism only occurs under anaerobic or microaerophilic conditions. Genome analysis of *A. thiooxidans *DSM 17138 showed that conserved genes involved in nitrate and nitrite assimilation as *narB *and *nirBD *are present. This evidence suggests that this bacterium has the ability to assimilate both nitrate and nitrite from the environment (Fig. 4). Nevertheless, experimental evidence under anaerobic conditions would be required to verify this.

#### Ammonia uptake

Ammonia transporters (Amt) have been described for almost all organisms, including bacteria [75], and catalyze the movement of ammonia across the cell membrane. We identified genes encoding ammonia permeases (*amtB*) in all three microorganisms examined in this study. However, assimilation of ammonia from the environment appears to be the only source of nitrogen for *L. ferriphilum *DSM 17947, consistent with that reported for *Leptospirillum sp*. Group II [2,3].

### Regulation of nitrogen assimilation

The process of nitrogen uptake, particularly nitrogen fixation, is energetically costly, and has therefore been shown to be tightly regulated [[18,19], and [25]]. To assess the regulatory mechanisms for nitrogen uptake in *A. ferrooxidans *DSM 16787, *A. thiooxidans *DSM 17318 and *L. ferriphilum *DSM 17947, we compared the genetic information from these three organisms to genomic information from microorganisms with previously described regulatory systems, focusing in particular on the genome of *A. ferrooxidans *DSM 16786, which we determined to be capable of nitrogen fixation.

#### Nitrogen fixation (NifA, DraTG)

**Figure 5:**
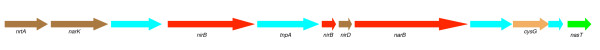
**Schematic diagram of the *A. thiooxidans *DSM17318 genomic region containing putative nitrate assimilation genes**. The following genes are indicated: *ntrA *encodes the periplasmic component of the nitrate transport system; *narK *encodes a nitrate/nitrite transporter; *nirB *encodes a nitrite reductase, which is interrupted by the transposase *tnpA*; *nirD *encodes the nitrite reductase small subunit; *narB *encodes a nitrate reductase; *cysG *encodes an uroporphyrin-III C-methyltransferase; and *nasT *encodes a nitrate assimilation system regulator.

**Figure 6:**
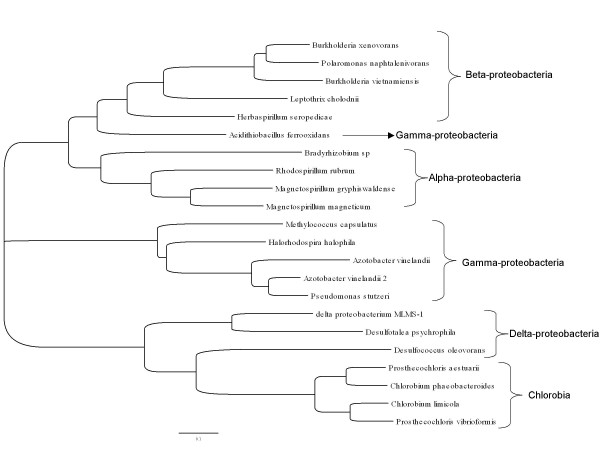
**Phylogenetic tree based on NifA protein sequences**. The tree was inferred using the Neighbor-Joining method, with 1000 replicates. Only those branches that appear in more than 50% of the boostrap replicates are considered. Evolutionary distances were computed using the JTT matrix. Analysis was conducted using the software MEGA4 [110].

For γ-proteobacteria, nitrogen fixation has been shown to be regulated via the NifLA system [18,19,76]. The NifA protein is a transcription factor that regulates the nitrogenase operon, whereas NifL provides post-translational inactivation of NifA when 2-oxoglutarate levels are high, indicating an excess of nitrogen in the cell [77]. In the γ-proteobacteria *A. ferrooxidans *DSM 16786, we identified a *nifA *gene near the nitrogenase operon (Figure 5), but did not find any *nifL *homologs, which suggests that the regulation of nitrogen fixation in these microorganisms is different from that previously described for other γ-proteobacteria. This was supported by a NifA-based phylogenetic analysis which grouped *A. ferrooxidans *DSM 16786 with members of the β-proteobacteria (Figure 6). This suggests horizontal transfer of this nitrogen regulation mechanism to *A. ferrooxidans *DSM 16786, similar to what has been proposed for other genes in this microorganism [78]. In addition, the absence of the *nifL *gene appears to be extended not only to the DSM 16786 strain, because we did not find this gene to be present neither in the genome sequence of the ATCC 23270 strain nor in the recently available genome sequence of the ATCC 53993.

The lack of *nifL *in the *A. ferrooxidans *DSM 16786 genome makes it difficult to discern the manner in which oxygen levels affect the regulation of nitrogen fixation in this microorganism. Nitrogenase is very sensitive to oxygen levels, and microorganisms cope with this problem via strategies such as anaerobiosis, high rates of oxygen consumption, and compartmentalization. In γ-proteobacteria, the NifL protein responds to high oxygen by inactivating the NifA protein, whereas in low oxygen conditions, the NifL protein activates the NifA regulator to stimulate expression of the nitrogenase genes [18,79]. It has been reported that in some microorganisms such as Rhizobia, which also lacks *nifL*, NifA directly responds to oxygen levels via an N-terminal cysteine motif (CXXXXC) [18,79]. We identified a similar N-terminal cysteine motif in the translated *A. ferrooxidans nifA *sequence, suggesting that this microorganism circumvents the need for NifL by utilizing NifA for direct response to oxygen levels.

In the *A. ferrooxidans *DSM 16786 genome, we also identified genes encoding dinitrogenase reductase-activating glycohydrolase (DraG) and dinitrogenase reductase ADP-ribosyltransferase (DraT) proteins near the nitrogenase operon (Figure 5). These gene products are involved in post-translational regulation of the nitrogenase complex in α-proteobacteria [18,80]. Under high nitrogen conditions, DraT inactivates nitrogenase via ADP-ribosylation [80]. Under low nitrogen conditions, DraG catalyzes removal of the ADP-ribose from nitrogenase to activate the enzyme [80]. Only recently, the presence of these genes was described in the ATCC23270 strain of *A. ferrooxidans *[5], making this microorganism the first reported γ-proteobacteria to be carrying such genes, again suggesting lateral transfer from other microorganisms.

Although nitrogenase genes were not identified in the *A. thiooxidans *DSM 17318 and *L. ferriphilum *DSM 17947 genomes, we searched these genomes for nitrogenase regulatory elements. In *L. ferriphilum *DSM 17947, we identified a candidate *nifA *homolog, annotated as a NifA-like transcriptional regulator, but the gene was located in a region containing genes involved in the synthesis of flagella. This NifA candidate has an 82% identity with the NifA transcriptional regulator from *Leptospirillum sp*. Group II. In *Leptospirillum sp*. Group II, *nifA *is also located in a region containing genes involved in the synthesis of flagella. It is possible that in *L. ferriphilum*, this NifA-like protein regulates the synthesis and/or the assembly of flagella, but this needs to be explored further. Of note, the NifA gene in *L. ferriphilum *DSM 17947 is distinct from that in *L. ferrooxidans*, an organism previously shown to participate in nitrogen fixation in the Tinto River [23,112], and from that in *L. ferrodiazotrophum*, an organism previously connected to nitrogen fixation in the acid mine drainage [24].

#### The NtrB/NtrC two-component system

Another important regulatory system in the assimilation of nitrogen compounds is the NtrB/NtrC (Part of the nitrogen regulation system Ntr) two-component system [18,19]. This system is involved in bacterial response to different nitrogen sources, such as molecular nitrogen, ammonia, or nitrate, and is present in great number of microorganisms [18]. The NtrB protein is a kinase, which activates NtrC via phosphorylation under low nitrogen conditions. NtrC is a transcription factor that not only regulates the *glnA *gene, which encodes the glutamine synthetase enzyme required for metabolic incorporation of ammonia, and the *glnK-amtB *operon, which encodes a PII regulatory protein and an ammonia permease protein, but also regulates its own operon (*ntrBC*). Positive regulation of *ntrBC *is triggered in response to low intracellular levels of glutamine via the PII sensory system [18,81,82]. We identified genes encoding proteins of the NtrB/NtrC two-component system in the *A. ferrooxidans *DSM 16786 and *A. thiooxidans *DSM 17318 genomes. In contrast, we identified genes from the NtrY/NtrX two-component nitrogen assimilation system in the *L. ferriphilum *DSM 17947 genome. NtrX is a transcriptional regulator similar to members of the Fis family (53% identity with a protein from *Geobacter uraniireducens*) whereas NtrY is a membrane-bound sensor kinase protein. The NtrY/NtrX system has been described in *Azorhizobium caulinodans *and *Azospirillum brasilense *(both diazotrophs), and in *A. brasilense*, where it was shown to participate in the regulation of nitrogen assimilation via detection of ammonia [83]. This suggests that in *L. ferriphilum *DSM 17947, NtrY detects ammonia levels and regulates the transcription factor NtrX accordingly.

Because *amtB *is present in all three organisms examined in this study, this suggests a common mechanism for regulation of ammonia uptake [18,84]. In *A. ferrooxidans *DSM 16786 and *A. thiooxidans *DSM 17318, ammonia levels are detected in response to glutamine concentrations and uptake is controlled by the NtrB/NtrC system. In *L. ferriphilum *DSM 17947, ammonia levels are directly detected by NtrY and uptake is controlled by the NtrY/NtrX system. Nonetheless, both systems likely control similar target genes.

#### PII protein family

The PII family of signal transduction proteins are found in eukarya, bacteria and archaea [81,82]. These proteins comprise one of the central mechanisms for controlling the metabolism of nitrogen and carbon in the cell [26,85] via detection of intracellular levels of different compounds, like glutamine, ATP, and 2-oxoglutarate [18]. This allows them to integrate the information from nitrogen (glutamine, ATP) and the carbon metabolism (ATP, 2-oxoglutarate), thus activating or inactivating several enzymes and transcription factors according to the requirements of the cell [18,19,26,81,82,85].

**Figure 7:**
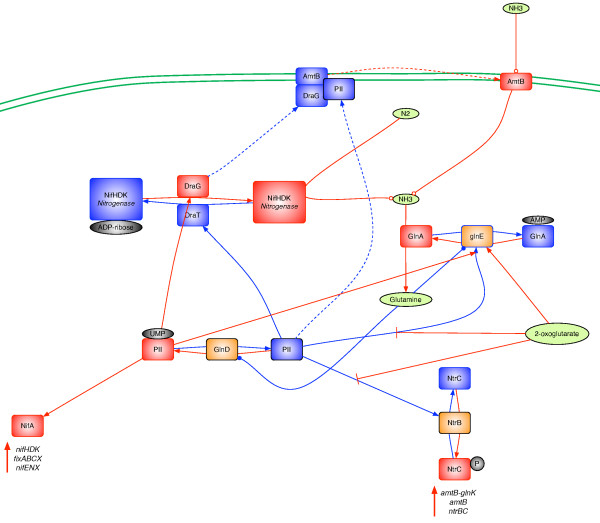
**Regulatory model of nitrogen assimilation proposed for *A. ferrooxidans *DSM 16786**. Proteins (boxes) and their resulting functions (lines) are indicated in red for activation during low nitrogen conditions and in blue during high nitrogen condition. In green are identified the principal effectors and nitrogen source that trigger the activation/inactivation of the enzymes involved in the model: ammonia (NH3), molecular nitrogen (N2), glutamine and 2-oxoglutarate. In orange are represented the signal transducer proteins: GlnD (uridylyltransferase), NtrB (Nitrogen regulation protein ntrB) and GlnE (Glutamate-ammonia-ligase adenylyltransferase). It is important to mention that this model considers the role of NifA in activating target genes under low nitrogen conditions.

We identified several members of the PII proteins family (See additional file 2: NitAsilProts.csv for the list of proteins) within the genomes of the three microorganisms examined in this study: *A. ferrooxidans *DSM 16786 had four PII family member genes, whereas *A. thiooxidans *DSM 17318 and *L. ferriphilum *DSM 17947 only had two. This difference may reflect the finding that only *A. ferrooxidans *DSM 16786 is capable of nitrogen fixation, thus requiring a tighter control over the process involved in nitrogen assimilation (nitrogen fixation and ammonia uptake). In Figure 7 we propose a model depicting the regulation of nitrogen assimilation in *A. ferrooxidans *DSM 16786. In this organism, the PII proteins are germane to regulation of nitrogen levels in the cell, the key effectors being glutamine and 2-oxoglutarate. Under low nitrogen conditions, PII protein is uridylylated by GlnD (uridylyl transferase), which is active when glutamine levels are low [81,82]. Uridylylated PII interacts with DraG protein to remove the ADP-ribose from nitrogenase, rendering this enzyme active for nitrogen fixation. Uridylylated PII also interacts with the NifA regulatory protein to activate NifA and stimulate the transcription of its target genes (e.g., the nitrogenase operon and the elements required for its assembly). In addition, when nitrogen levels are low, the intracellular levels of 2-oxoglutarate are high [81,82], which inactivates the action of the non-uridylylated PII protein, resulting not only in the removal of AMP from glutamine synthetase (GlnA) to stimulate glutamine synthesis from ammonia but also in stimulation of NtrC phosphorylation by NtrB to induce the transcription of several targets including the *amtB-glnK *operon, which contains an ammonia permease and a PII protein, and the *ntrBC *operon itself [18,19]. Under low nitrogen conditions, AmtB permease participates in the uptake of ammonia, which is transformed into glutamine by glutamine synthetase (GlnA). Under high nitrogen conditions, glutamine stimulates GlnD, which removes the uridylyl moiety from the PII protein [81,82]. This protein stimulates DraT, which inactivates nitrogenase by ADP-ribosylation. Also, the non-uridylylated PII protein sequesters DraG to the membrane and binds to AmtB permease, blocking the uptake of ammonia into the cell [18,80]. In addition, because 2-oxoglutarate levels are low, the non-uridylylyated PII protein stimulates NtrB dephosporylation of NtrC to prevent activation of NtrC target genes. Non-uridylylyated PII also stimulates GlnE, which inactivates glutamine synthetase by AMP-ribosylation [18,19]. We propose that *A. ferrooxidans *likely has four PII genes because both nitrogen fixation and ammonia uptake mechanisms are in operation. The PII family of proteins is also central for the other two organisms examined in this study. However, because *A. thiooxidans *DSM 17318 utilizes nitrate reduction and ammonia uptake and because *L. ferriphilum *DSM 17947 utilizes only ammonia uptake, only two PII genes are required to carry out nitrogen metabolism. For *A. thiooxidans *DSM 17138 and *A. ferrooxidans *DSM 16786; the common elements of ammonia uptake are those involved in detection and regulation of ammonia levels. Similar to nitrogen fixation, nitrate assimilation likely occurs under low oxygen conditions, is tightly regulated, and involves not only detection of glutamine and 2-oxoglutarate levels, but also detection of oxygen levels, for expression of the appropriate genes. Further exploration is needed to establish the connection between ammonia uptake and nitrate assimilation in *A. thiooxidans*.

An unexplored issue for nitrogen fixation in *A. ferrooxidans *is the oxygen sensitivity of the nitrogenase enzyme. *A. ferrooxidans *typically uses oxygen as its terminal electron acceptor during nitrogen fixation, although it reportedly grows under anaerobic conditions using a ferric ion as the terminal electron acceptor [86]. We have recent evidence that this nitrogenase enzyme is functioning under aerobic conditions on some substrates (data not shown). We believe that in *A. ferrooxidans*, inactivation of nitrogenase may be prevented by two parallel mechanisms: increased activity of the terminal cytochrome oxidase bd protein may impart respiratory nitrogenase protection [87] and a ferrodoxin "FeSII"-like protein may provide conformational protection of nitrogenase. Both protective mechanisms have been described for *Azotobacter vinelandii *[88,89]. A homolog of the *A. vinelandii *FeSII gene was identified two open reading frames downstream of the nitrogenase gene *nifK*, and would likely be co-expressed with the complete *nif *operon (data not shown). This putative ferredoxin protein may protect nitrogenase from irreversible inactivation mediated by oxygen, as has also been described for *Gluconobacter diazotrophicus *[90]. More experimental data is necessary to confirm this hypothesis.

Although this study provides important genetic information regarding *L. ferriphilum *DSM 17947, there is insufficient information for development of a nitrogen metabolism regulatory model. For example, a possible interaction between the NtrX/NtrY system and the PII proteins has been recently explored in *Rhodobacter capsulatus *[91]; for *L. ferriphilum *DSM 17947, it is unclear which nitrogenous species is detected by NtrX/NtrY. In addition, other *Leptospirillum *species [4,24] are reportedly capable of nitrogen fixation, in contrast to *L. ferriphilum*, making extrapolation from one species to another difficult.

Recently it has been reported the genome sequence of another strain of *Acidithiobacillus ferrooxidans*, which was released by the Joint Genome Institute and named as *A. ferrooxidans *ATCC 53993. This strain was previously characterized as *L. ferrooxidans*, but now with the genome available it has been reclassified as a member of the *Acidithiobacillus *genus. Comparing the genome sequence of the ATCC 53993 strain and our *A. ferrooxidans *DSM 16786 strain, we observed some differences related with the genome organization (manuscript in preparation), but the genes and mechanisms involved in carbon and nitrogen fixation that we are discussing in this paper, are also present in the ATCC 53993 strain. A similar situation occurred when we compared the genome sequence of our *L. ferriphilum *strain DSM 17947, against the genome sequence of the microorganism *Leptospirillum *sp. Group II UBA (92), where the same genes that we described in this work for the carbon and nitrogen assimilation, are also present in this microorganism, confirming that our findings are conserved in microorganisms from the same specie.

Based on our genomic analysis, an ecological role for each of these three microorganisms within a bioleaching community can be proposed. In this type of environment, energy is not the limiting factor for the development of the microbial community, as sulfur or iron minerals are abundant and can be used as electron donors. Therefore, carbon and/or nitrogen are likely the most limiting elements for the development of the microbial biomass. Given this limitation, *A. ferrooxidans *DSM 16786, or other nitrogen fixing microorganism, may act as the primary supplier of nitrogen (either in the form of nitrate or ammonia) and may therefore be essential for the establishment of a microbial community in this system.

Although each of the strains analyzed in this study is capable of carbon fixation, different pathways are employed to accomplish this purpose. The Calvin-Benson-Bassham cycle represents the most important extant autotrophic carbon fixation pathway. Despite its global significance, it is restricted to organisms with high-energy yield from a chemotrophic or phototrophic lifestyle. Microorganisms present in extreme environments (e.g., high temperature, anaerobic, or acidic conditions) generally utilize different CO_2 _fixation pathways [37]. Thus, the presence of the RTCA cycle in *Leptospirillum *reflects the fact that these microorganisms are more metabolically restricted than *A. ferrooxidans *or *A. thiooxidans; Leptospirillum *is the only genus which strictly uses ferrous iron as an electron donor. Additionally, the presence of two completely different CO_2 _fixation mechanisms in the *Acidithiobacillus *and *Leptospirillum *genera likely reflects the distinct growth and colonization capacities of these bacteria in extreme environmental conditions. This might also explain the dominance of the *Leptospirillum *genus reported in bioleaching communities from industrial operations or from naturally extreme environments [93,94].

To date, a lack of mutational studies and/or knockout strains has prohibited analysis of CO_2 _and nitrogen metabolisms in *A. ferrooxidans*, *A. thiooxidans*, and *L. ferriphilum*. However, as demonstrated here and elsewhere [3,24], genomic and other global (transcriptomic and metatranscriptomic) approaches (1,4) can bypass this limitation to provide relevant information regarding individual and community metabolisms.

## Conclusion

The genomic study presented here, is the first attempt to describe the metabolic tactics used by a community of three chemolitotrophic bacteria found in a Chilean biomining environment. *Acidithiobacillus ferrooxidans *is capable of oxidizing iron and sulfides as energy source, whereas *Acidithiobacillus thiooxidans *only oxidizes sulfides and *Leptospirillum ferriphilum *only oxidizes iron. These three organisms often share the same environmental niche, but their relative abundance differs depending on whether their surroundings are natural or modified by operations such as mining, likely because the nutrient sources in both cases are completely different. To have a deeper insight on how microorganisms take advantage of the CO_2 _and nitrogen resources present in their environment we performed this comparative analysis concluding the following main facts:

*A. ferrooxidans *fixes CO_2 _by the Calvin-Benson-Bassham (CBB) cycle, the same mechanism used by *A. thiooxidans*, but the latter has only 1 copy of Rubisco type I instead of the 2 copies found in *A. ferrooxidans*. A different and novel situation was discovered for *Leptospirillum ferriphilum *that is fixing CO_2 _by the reductive tricarboxylic acid cycle.

With respect to nitrogen source assimilation we discovered that while all the species analyzed can incorporate ammonia by their ammonia transporter, *Acidithiobacillus thiooxidans *can assimilate nitrate and nitrite and only *Acidithiobacillus ferrooxidans *is able to fix nitrogen directly from the air

## Methods

### Strains and culture conditions

*A. ferrooxidans *DSM 16786, *A. thiooxidans *DSM 17318 and *L. ferriphilum *DSM 17947 (all strains owned by BioSigma SA) were grown at 30°C with shaking (200 rpm) in basal 9 K medium ((NH_4_)SO_4_: 0.4 g/L, K_2_HPO_4_: 0.4 g/L, MgSO_4_-7H_2_O: 0.4 g/L) adjusted to pH 1.8 with concentrated sulfuric acid and supplemented with i) FeSO_4_-7H_2_0: 30 g/L, ii) S°: 1% w/v and iii) FeSO_4_-7H_2_0: 15 g/L plus Fe_2_(SO_4_)_3_: 12,4 g/L, respectively. Cell number was determined by chamber counting under microscope (Thoma Chamber, depth 0.010 mm). Cultures were harvested by centrifugation at 12,000 × *g *for 20 min at 4°C.

To evaluate the growth of *A. thiooxidans *DSM 17318 on nitrate, cells were grown in 9 K medium with or without ammonia and supplemented with 0.5–4.0 g/L KNO_3_, above described. Nitrate and nitrite concentrations were quantified using Nitratest (Merck) according to manufacturer's instructions.

### Generation of Genomics Library and Sequence Analysis

Sequencing of *A. ferrooxidans *DSM 16786 performed using a shotgun library of 5,568 clones (2,000 bp each) sequenced by Seqwrite (Houston, TX) and a second library of 1,433 fosmids (40,000 bp each) sequenced by Agencourt (Boston, MA), with an estimated coverage 2.79 folds. The final assembly contained 764 contigs forming 286 scaffolds; the biggest scaffolds covered 62% of the total sequence, while the general coverage of all the assembled contigs is estimated to be near a 94% of the total sequence. The genome of *A. thiooxidans *DSM 17318 was sequenced using a shotgun library of 18,0480 plasmid clones (4,000 bp each) and 11,088 fosmid clones (40,000 bp each) sequenced by Agencourt (Boston, MA), with an estimated coverage of 4.5×. The final assembly contained 882 contigs forming 283 scaffolds; the seven biggest scaffolds covered the 75% of the sequence, while the general coverage of all the assembled contigs is estimated to be near 92% of the total sequence. The genome of *L. ferriphilum *DSM 17947 was sequenced using a shotgun library 13,645 plasmid clones (2,000 bp each) and 10,088 fosmids clones (40,000 bp each) sequenced by Agencourt (Boston MA), with an estimated coverage of 5.47×. The final assembly contained 321 contigs forming 89 scaffolds; the five biggest scaffolds covered 87% of the sequence, while the general coverage of all the assembled contigs is estimated to be near a 94% of the total sequence. For all the three genomes, base calling was performed using Phred [95,96] and resulting reads were assembled using a two-stage method: contigs were formed using Arachne [97], consensus sequences of each contig were obtained using Phrap [98]. Scaffolds were formed using the Bambus software [98] based on read mate-pairing. Ambiguities were solved by comparison to the reference sequences and by manual curation.

### Genome annotation

The assembled sequences of the three genomes were annotated using the GenDB annotation system [100]. Candidate ORFs were marked using Glimmer/Critica [101] and annotated by homology to the COG database [102], to non-redundant proteins from NCBI and to previously described proteins from the literature. Protein domains were marked using InterPro [103]. Afterwards automatic annotation was manually cured.

### PCR and RT-PCR

Reverse transcriptase PCR (RT-PCR) was carried out in order to identify co-transcribed genes of *L. ferriphilum *DSM 17947. PCR reactions were carried out to characterize the *nirB *gene of *A. thiooxidans *DSM 17318. For RT-PCR, total RNA was isolated from cells in late exponential phase using the protocol described in [104] Briefly, cell pellets were washed with a solution of 10 mM H_2_SO_4 _(pH1.2) followed by PBS buffer (pH 1.2) and suspended in Tris buffer (pH 8.0) containing EDTA, SDS, Triton X-100, and Tween 20 (STT buffer) [105]. The suspension was treated with proteinase K and phenol: chloroform extracted. The total RNA was precipitated using isopropanol as described in [105]. Genomic DNA for PCR amplification of *nirB *was obtain using the same protocol in [104], for total RNA extraction described previously. PCR and RT-PCR were carried out by standard procedures and included various control reactions that accompanied each experiment. The DNA sequences of the various primers used for both RT-PCR and PCR and their locations on the genomic open reading frame context are provided (See additional file 3: OligoList.pdf for the list of primers used).

### Bioinformatics sequence analysis

The genomic sequences from the three microorganisms under investigation were examined. Proteins involved in known carbon fixation pathways were obtained from the KEGG database [106]. Amino acid sequences derived from genes identified as being involved in the Calvin cycle and the RTCA cycle were used as query sequences to search the translated nucleotide database from the genomes of the *A. ferrooxidans *DSM 16786, *A. thiooxidans *DSM 17318 and *L. ferriphilum *DSM 17947 strains using tBlastn [107] with default parameters. When a prospective candidate gene was identified, its predicted amino acid sequence was used to formulate a BlastP [107] search of the NCBI non-redundant data base. Only the best hits were accepted as evidence for putative orthologs. Candidate genes and their corresponding translated proteins were further characterized using the following bioinformatics tools: primary structure similarity relations were determined using ClustalW 1.8 [108], structural motif predictions were determined using Prosite [109] and peptide domain predictions were determined using ProDom [110].

Phylogenetic analyses were performed using the Mega software version 4.0 [111]. Tree drawing and visualization was done using the software FigTree 1.1.2 [112].

## Authors' contributions

PP and AM designed the research. GL, JAU and NE performed the experimental and bioinformatics research and analyzed data. GL, JAU, NE and PP prepared the manuscript.

## Supplementary Material

Additional file 1 **Genes involved in carbon fixation in *A. ferroxidans *DSM 16786, *A. thiooxidans *DSM 16786 and *L. ferriphilum *DSM17947.** Click here for file

Additional file 2 **Genes involved in nitrogen assimilation in *A. ferrooxidans *DSM 16786, *A. thiooxidans *DSM 16786 and *L. ferriphilum *DSM 17947.** Click here for file

Additional file 3 **Oligonucleotide primers used for RT-PCR amplification reactions.** Click here for file
