# Supplementary material for: Comparative genomic analysis of carbon and nitrogen assimilation mechanisms in three indigenous bioleaching bacteria: predictions and validations
Source: BMC Genomics. 2008 Dec 3;9:581. doi: 10.1186/1471-2164-9-581 (PMC2607301; doi:10.1186/1471-2164-9-581)
Supplement: Additional file 3 — Oligonucleotide primers used for RT-PCR amplification reactions. [file 1471-2164-9-581-S3.pdf]

Oligonucleotides used for RT-PCR reactions

Number	Sequence	Target gene
1	AGCCGGAAGTTGTCGCATAAT	*ccsA*
2	GCCCACAGACGGAACATAGCA	*ccsB*
3	CCGGTCAAGGTGGCGTTTTACTAC	*ccsB*
4	CTGCGTCAAGGTCTGGTCAATCT	*acnA*
5	CCGCCCTTGCCCCGATGTA	*acnA*
6	AGATAGCCTCCGCGAAACTGAC	*ccl*
7	GGCGCGATCGAGCAGTGTG	*ccl*
8	CCAGTGCATCCTTCTGCAC	*orf1*
9	CCGGACTGTCGTGGCCTACTTTA	orf1
10	ACCTGCCATTCCTTGCCGTTGAT	*fdrB*
11	TGTCATGTGCGGTCTCTGTGTT	*fdrB*
12	GAATGACCTCCCCCTTGTTGG	*fdrA*
13	TTGCTGGAAGTGTTTGTCTACG	*fdrA*
14	GCTTCCGGGCCAGTTTTA	*sucC*
15	GGTGGCGGAAGAGCTGTTTGAG	*sucC*
16	TTTGCCCCGTCTGGAGTAATGA	*sucD*
17	TGCCGGCGCCATTGTTT	*sucD*
18	GCCTTGTCTCCCACTTTTATTTCA	*trx*
19	GACCCGGGAATTGCGTGAAGC	*forA*
20	TCGTGAGCCGGATAAAAAGTGTC	*forB*
21	GCGGTCATGCATTCCTGGTTC	*forB*
22	GACTTGCCGTGCGTAATCACC	*forG*
23	TCGGAAAGTATAAGGAGACAAG	*forG*
24	TCAAAAACATCAACCAGAAGAC	*orf2*
25	TGCGTCAGGGGAAAAAGGTAT	*orf2*
26	TTCCCGCTCAATCTGGTTCTG	*orf3*
27	GGTTGAGCAGGGGGAGAATG	*orf3*
28	ATGGGGTATGCGACGGAAAAG	*porA*
29	GCCCCCGATCGGTTCAGTCAA	*porA*
30	TCACCGGCTCAAACGAT	*porB*
31	ATGGCGCCGGTGGGAAAGAAAT	*porB*
32	GGGCTTCCATACCAATAACATCTG	*porG*
33	TATGTTCCCGCGACTAAGATG	*porG*
34	TGGCCTCCGGACAATACA	*porE*
35	GTACAATGTTGCAGAAATCAGTG	*porE*
36	TCGCTGACCAGTTGCCCTTC	*porD*
